# Vocal imprecision as a universal constraint on the structure of musical scales

**DOI:** 10.1038/s41598-022-24035-6

**Published:** 2022-11-17

**Authors:** Elizabeth Phillips, Steven Brown

**Affiliations:** Department of Psychology, Neuroscience & Behaviour, McMaster University, 1280 Main St. West, Hamilton, ON L8S 4K1 Canada

**Keywords:** Evolution, Psychology

## Abstract

Theories of the origin of musical scales from the ancient Greeks to the present day have assumed that the intervals comprising scales are defined by specific mathematical ratios. Such theories are predicated on pre-tunable instruments, and yet *the voice* is almost certainly the original musical instrument. Therefore, the analysis of vocal scales offers a more naturalistic approach to understanding the origin of musical scales. In the present study, we conducted a large-scale computational analysis of vocal pitch-class properties and their implications for scale structure. We analyzed 418 field recordings of solo, unaccompanied songs from across 10 principal musical-style regions of the world. The results revealed a mean vocal pitch-class imprecision of approximately 1.5 semitones, and comparable results were obtained across all regions. These results suggest that vocal imprecision is universal and is mainly derived from the physiological limitations of the voice. Such vocal imprecision fundamentally constrains the formation of musical scale structure: it provides a lower limit on the spacing between adjacent scale tones and thus an upper limit on the number of scale tones that an octave can contain. We discuss these results in terms of an Interval Spacing model of the evolution of musical scales.

## Introduction

One of the oldest ideas about the origins of music in Western culture is that musical intervals can be mathematically described as simple integer ratios between the lengths of vibrating strings. This idea has served as the foundation for theoretical accounts not just of musical intervals, but of musical scales more generally. A dominant theory of the origin of diatonic Western scales argues that they are entirely derived from simple integer ratios between the scale degrees’ acoustic frequencies (e.g., 2:1 for the octave, 3:2 for the perfect fifth, 4:3 for the perfect fourth, and so on)^1^. In 1722, Rameau affirmed that harmonics were the ultimate basis of scale structure: “a knowledge of harmony is sufficient for a complete understanding of all of the properties of music” (p. 3)^2^. We will consider this viewpoint as the Harmonicity theory of the origin of scales, and it has become the established model within music psychology and psychoacoustics. As Kreitler and Kreitler describe it, scales are derived from the “overheard overtones” (p. 129)^3^ assumed to be perceivable in the harmonic series of single pitched sounds, most especially speech sounds^4^. The idealized scales of Harmonicity theory use tuning systems of “just intonation,” in which all of the scale degrees are defined as simple integer ratios of one another.

Whereas Harmonicity theory is a top-down theory of scales based on optimizing mathematical simplicity, an alternative bottom-up model is grounded in the physiology of vocal pitch production^5,6^. The voice is the oldest and most universal musical instrument, and thus vocal constraints on the structure of musical scales are of utmost importance in thinking about the evolution of music. In fact, vocal music has been a highly neglected source of information in the study of musical scales. Perhaps the most agreed-upon feature of vocal music is the imprecision in pitch production. Numerous studies have found that the voice is highly imprecise as a pitch-generating instrument, especially compared to tunable instruments like violins and flutes^5,7^. While synthesizers can produce the exact frequency dictated, acoustic instruments produce pitch- and interval-classes with a distribution of frequencies surrounding the target pitch^8–10^. In the case of the voice, such a pitch distribution often spans at least a semitone (100 cents) on either side of the target frequency^5^. Wide distributions such as these have been described as “interval islands”, analogous to the “vowel ellipses” of vowel sounds in phonetics^11–14^. This observation has led to the formulation of a voice-based model of the origin of musical scales—called the Interval Spacing theory^5^—that posits that the intrinsic imprecision of vocal production constrains the spacing of pitch-classes in musical scales. Adjacent pitch-classes must be far enough apart to be distinguishable from one another, but not so distant as to hinder accurate singing^6^.

Evaluating whether Harmonicity theory or Interval Spacing theory better accounts for the nature of musical scales requires the analysis of a culturally diverse sample of music and the development of an empirically descriptive model thereof. Most theories of musical scales have been predicated on the prescriptive tuning regimens of various art and classical musics, including systems of theoretical modes and instrumental tunings^15,16^. Such an approach allows scales to be predefined in an abstract sense, but without any reference to actual melody production. The approach thus hinders an investigation into fundamental issues surrounding the origin of musical scales. Does melody precede scale or vice versa? How do scales arise in vocal music given that the voice cannot be pre-tuned?

These questions can only be addressed by taking a descriptive and comparative approach to the study of musical scales, particularly in indigenous and traditional vocal songs. In comparative linguistics, researchers construct vowel inventories by analyzing the way that people actually speak. By analogy, comparative musicologists need to construct models of scale structure by analyzing *the way that people actually sing*. This is not a new idea. Meyer discussed a similar approach when he stated that "scales are not the raw material of melodies. It is just the other way around: melodies are the materials from which scales are abstracted” (pp. 216–217)^17^. The overall approach of the present study is to move beyond the study of abstract instrumental tunings in art music traditions and instead investigate the actual production of melodies in indigenous and traditional vocal music, and do so at a global level that accounts for the diverse panoply of scale types and singing styles worldwide.

A few previous studies^4,18^ have recognized the evolutionary significance of the human voice and have posited that its harmonic features might account for scale structure. However, the explanatory power of such models for the origin of musical scales has been questioned^6,19^, largely because they fail to account for the spacing between scale degrees and thus melodic intervals. Other recent comparative musicology studies have analyzed cross-cultural features of melodies^20,21^ and scales^22,23^, and some have looked at specific pitch-class features, including how these features relate to scale structure, although within a relatively small sample^24–27^. However, there has yet to be a globally-comprehensive, computational survey of the structural features of pitch-classes using a worldwide database of recorded vocal melodies. Such a study is necessary to understand how pitch-production constraints might influence how scales are structured and used, which is itself necessary in order to develop a generalizable, descriptive model of musical scales, and eventually to investigate its ties to physiological, cognitive, cultural, and evolutionary mechanisms.

In the present study, we constructed a large, culturally-diverse database of 418 solo, monophonic vocal song recordings, focusing exclusively on indigenous and traditional musics. We attempted to represent music from across 10 principal musical-style regions of the world, as characterized in Alan Lomax’s Cantometrics project and as presented in the book *Folk Song Style and Culture*^28^. For each song, we extracted the fundamental frequencies of the melody using Tarsos^29^ and then measured two dependent variables for each pitch-class: *imprecision*, corresponding to the range of the pitch-class’s frequency distribution in cents, and *inaccuracy*, corresponding to how well (or poorly) the tuning conformed to the mathematical template given by twelve-tone equal temperament (12-ET), also in cents. We analyzed global trends for both variables and looked for cross-cultural differences. Because indigenous vocal music is not typically studied in Western research, we also wanted to compare these results to those obtained from a more-typical sample of recordings, while controlling for melodic features that may impact pitch production. To do so, we carried out a small experimentally-controlled study in which a common set of eight melodies from the database was performed by trained Western musicians on four different instruments that vary in their degree of pre-tuning, namely an organ, flute, trombone, and voice. We again measured pitch-class imprecision and inaccuracy in order to determine whether these variables differed across the instruments as a function of the fixedness of their tuning.

We generated three major predictions for the overall study. First, if vocal imprecision is a universal physiological constraint for melody production, then it should occur at a comparably high level in all cultures, with pitch-classes being minimally 100 cents wide. This would be supportive of the Interval Spacing theory. However, if imprecision is low in some or all cultures, or if it is variable across cultures, then this would oppose the theory, in which case the role of enculturation on pitch production and scale structure would need to be further explored. Second, if Harmonicity theory is a valid tuning theory not just for Western instrumental art music but for traditional vocal musics worldwide, then pitch inaccuracy should occur at a comparably low level across all cultures, indicating that people’s sung pitches conform well with the analyzed tuning model. However, if inaccuracy is high in all cultures, then this would call into question scale theories based on mathematically-optimized notions of tonality. Third, if pitch-class imprecision and inaccuracy depend on the degree of pre-tuning of an instrument, then they should vary significantly between the organ, flute, trombone, and voice in the experimental study. This would suggest that prescriptive scale models based on theoretical modes or instrumental tuning systems do not suffice in describing acoustic music, where pitch is categorical and context-dependent. If so, newer descriptive scale models would need to be developed based on the analysis of actual melody production, whether that music be vocal or instrumental, Western or non-Western.

## Methods

### Study 1: global song analysis

#### Sample

**Figure 1:**
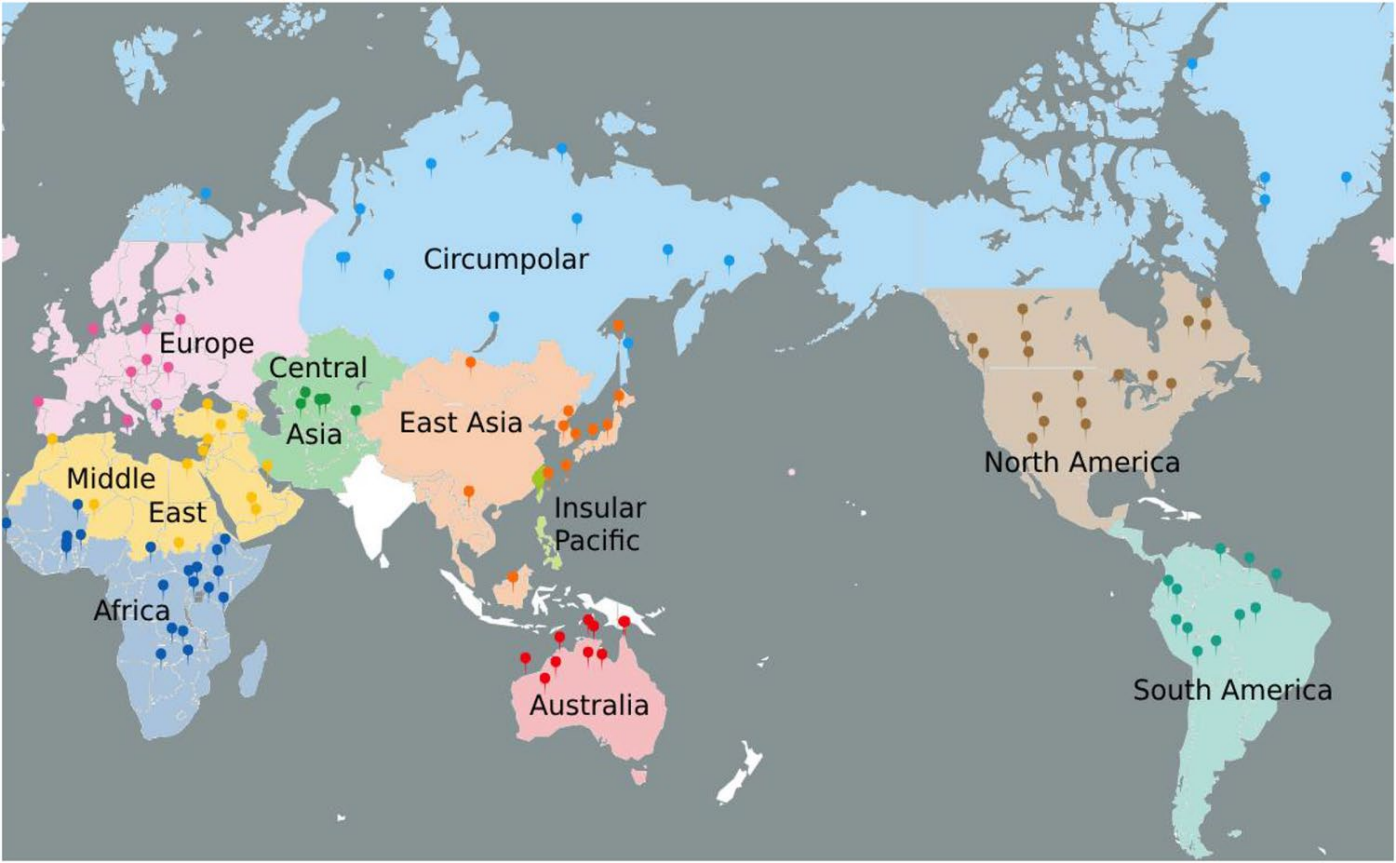
A geographic map of the song sampling according to the 10 major musical-style regions of the world, as color-coded by region. The 418 samples are distributed across style-regions as follows: Africa, 41 songs; Australia, 17; Central Asia, 16; Circumpolar region, 52; East Asia, 50; Europe, 51; Insular Pacific, 52; Middle East-North Africa, 38; North America, 50; South America, 51. The map was made in ArcGIS with further editing in Photopea.

We constructed a global database of 418 solo, unaccompanied, monophonic field recordings of vocal songs. We obtained these recordings from various private ethnographic collections, independent record labels and, most significantly, the Smithsonian Folkways record label. We restricted our sampling to indigenous musical traditions as much as possible, supplementing them with folk traditions where appropriate, but avoiding art traditions. Within this focus, we aimed to maximize the geographic diversity of our database while avoiding overly sparse sampling of any one culture. The geographic sampling aimed to represent songs from each of the 9 musical-style regions characterized by Lomax in his global analysis of song style^28^, except for an isolate called “Tribal India”, due to an inability to find recordings from this region. Because Lomax’s designation of “Old High Culture” covers an extremely large expanse of geography, and primarily characterizes the art music traditions therein, we divided it into three smaller, more traditional regions: Middle East-North Africa, Central Asia, and East Asia, resulting in 10 final regions. In addition, we opted, based on the terminology in Savage et al.^30^, to use the term “Circumpolar” to describe what Lomax described as “Arctic Asia” since this style-region includes the arctic part of North America as well (e.g., Inuit). Figure 1 presents a geographic map of the song sampling, including a listing of the number of samples analyzed for each stylistic zone.

For each song in the database, we recorded the following metadata: source collection; copyright owner; cultural group; recording year (if known); album title; song title; and song length. Some samples originally included in the database were excluded from analysis if the recordings were too noisy or had audio issues; if the pieces contained significant accompaniment, had no solo sections of sufficient length, or were sung by a child; or if the pitch-tracking algorithm could not produce sufficient fundamental-frequency annotations for quantitative analysis. We aimed to analyze 50 samples per region, although the limited availability of songs from certain regions led the final number of analyzed samples to be 418, rather than 500, for the 10 musical-style regions (see Fig. 1).

#### Scale analysis

**Figure 2:**
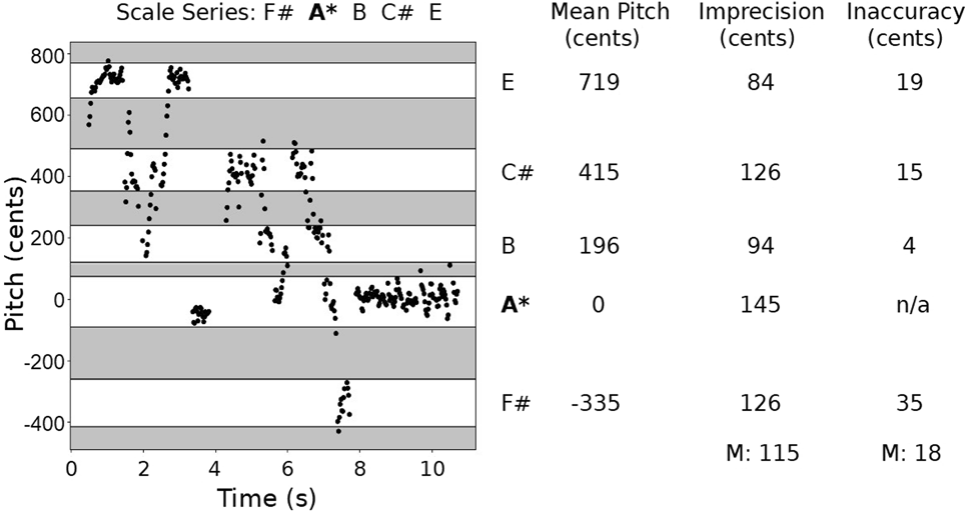
An example of a scale analysis for a song excerpt. Left: A melograph showing the pitch annotations for the ~ 11 s sample that was selected from *Chant a cappella*, a Circumpolar (Orok) melody from Siberia. The melograph has been segmented into pitch-classes using horizontal boundary lines, as shown by the white regions. Non-discrete pitch-classes and noise annotations have been greyed out, indicating that they were not analyzed. The y axis of the melograph lists the pitch values in cents relative to the tonic pitch, which has a value of 0. Right: The scale series and pitch-class analysis of *Chant a cappella,* indicating the five discrete pitch-classes by note name, mean cent value relative to the tonic, the range of each pitch-class in cents as a measure of imprecision, and the tuning inaccuracy in cents relative to 12-tone equal temperament. At the bottom of the Imprecision and Inaccuracy columns are the mean values for this sample, as indicated by M. In the scale sequences listed above and to the right of the melograph, the asterisk shown for pitch-class A signifies that this is the tonic pitch of the scale. A* has an “n/a” value for inaccuracy because it is the tonic pitch and thus the tuning reference.

We identified by ear major structural sections of the melodies, including repeated verses and points of considerable pitch shift or modulation, such as a sudden change in the set of pitch-classes used. We then chose a short excerpt (mean: 20.5 s; SD: 8.52) that contained at least one complete melodic idea for further analysis, but that did not contain significant pitch drift, such as the singer becoming gradually sharp or flat. For each melodic excerpt, we identified the scale series by ear. Scale series were notated using the closest possible Western alphabetical notes, from the lowest to highest pitch. The perceived tonic was indicated with a star. When the sung notes occurred in between keyboard notes, this was indicated with a slash (e.g., Eb/E). Non-discrete, unstable, and/or uncertain notes were indicated using parentheses. Secondary tonics, if present, were indicated using a double star. Figure 2 presents an example of such an analysis.

#### Quantitative analysis

We analyzed each song using Tarsos^26^ and its built-in YIN pitch-tracking algorithm^31^. After a pitch-trace was created, we used the “annotations visualizer” to select a melodic excerpt (time) and the salient range of annotations (pitch). These raw data were used for analysis in Jupyter Notebook^32^. All of our pitch analyses were carried out in cents, where 100 cents = 1 equal-tempered semitone. The pitch annotations for an excerpt were ordered from the lowest to highest value in cents, and the first derivative was calculated. The peaks of this function were then superimposed onto a melograph of the annotations to visualize initial estimations of the pitch-class boundaries. The boundaries were then adjusted by hand using the melograph as a visual reference. An example of a segmented melograph is shown on the left side of Fig. 2. When the final pitch-class boundaries were selected, they were used to bin the cent annotations into discrete pitch-classes. The tonic pitch was identified by ear and validated through inter-rater review (see below). The other pitch-classes were set relative to the mean of the tonic pitch-class, which was assigned a value of 0, where negative cent values referred to pitches below the tonic pitch. Non-discrete pitch-classes and noise were removed.

Two dependent variables were derived from these data, relating to the imprecision and tuning inaccuracy of the vocal production, respectively. For imprecision, we calculated the range of each pitch-class in cents by subtracting the minimum cent value from the maximum value. The range therefore corresponded to the width of the pitch-class (in cents). In order to constrain this variable, we considered values greater than 400 cents (+ /− a whole tone [200 cents]) as outliers and removed them from the analysis. Outliers were uncommon (n = 13), and we confirmed that a less-sensitive measure of variability (inter-quartile range) showed a similar patterns of results. To measure how accurately the pitch-classes conformed to a mathematical model of tuning (12-ET in this case), we calculated the absolute distance of each pitch-class’s mean cent value from the nearest 100-cent interval from the tonic, where the tonic marks 0 cents. This variable was constrained to be a positive value less than 50 cents, since a value of 50 cents marks the halfway point between any two adjacent pitch-classes in 12-ET. Values near 0 would indicate a close adherence to 12-ET, whereas larger values would indicate greater levels of tuning inaccuracy relative to this mathematical template. The right side of Fig. 2 presents values for both of the dependent variables for a sample song.

#### Inter-rater review

Three raters independently analyzed each song for its time-segments, scale series, tonic pitch, and pitch-class boundaries. The amount and type of disagreement were recorded. Consensus was reached by listening to the piece together and discussing each point of disagreement. Occasionally, if two analysts failed to come to an agreement, the third analyst would act as the tie-breaker. The results of the final agreed-upon analysis are presented here.

### Study 2: instrumental pitch production

#### Samples

This study was an exploratory experimental analysis that aimed to compare the voice with three Western aerophones (i.e., instruments for which air is the primary sound source). In particular, we wanted to investigate how the relative tuning fixedness of the various instruments would influence their pitch-class imprecision and inaccuracy. The instruments were selected to create a gradient of fixedness: organ > flute > trombone > voice. We predicted that pitch-class imprecision would negatively correlate to the degree of fixedness, with the voice being the least fixed and thus least precise. Eight short melodies were randomly chosen from our database (each from a different region) and transcribed into Western musical staff notation by the first author, with review by the other analysts. These melodies reflected a diverse sample of global musical styles, tonal structures, and melodic structures. The musical scores are available at the OSF repository for this study (see “Ethical approval”).

#### Participants

Three semi-professional musicians—a flutist, a trombonist, and a vocalist—were recruited via personal connections; the first author served as the organist. The mean age of the participants was 25 years old (SD = 1), and the mean experience at their instrument was 17 years (SD = 3). Participants were remunerated $100 (Canadian), with the exception of the first author. After consultation with the Research Ethics Board (REB), it was determined that the musicians were only being hired to assist with sample preparation, and as such were not considered as participants under the REB’s protocol. We followed pre-approved Covid safety measures during the on-campus and in-person recording session.

#### Methods

The musicians were sent the sheet music for the eight transcribed vocal songs several months in advance of the experiment. They reported spending an average of 6 h (SD = 5.3) practicing the music before the recording session. They were told to transpose each piece up or down in octaves as needed to place the melody within their comfortable register. They were instructed to perform all pieces with neutral musical articulation, a minimum of expression and vibrato, and to pay particular attention to tuning and pitch accuracy. The singer was instructed to sing the melodies using a single neutral vowel. Before each piece, the initial note was played on the organ, and the starting tempo was played on a metronome.

The musicians were recorded individually in a university concert hall, all on the same day. They performed all eight pieces in a different semi-randomized order. They were allowed to do a second recording of any piece if they desired and then inform us of which version to analyze. Recordings took approximately 30 min per musician. The recordings were made on an Audio Technica 4040 Caridoid Large Diaphragm Condenser Microphone placed 2 m in front of the performer’s sound source, using a Focusrite Clarett 2Pre USB audio interface at 24-bit depth and 96 kHz sampling rate. After the recording session, participants filled out a brief questionnaire in which they rated the perceived difficulty of the melodies, as well as an assessment of their own pitch imprecision and inaccuracy. Quantitative analysis of the recordings was carried out exactly as in Study 1 to obtain measures of pitch-class imprecision and inaccuracy.

### Ethical approval

No human participants were involved in this study. All protocols were carried out in accordance to Canada’s Tri-Council Policy Statement.

## Results

### Study 1: global song analysis

#### Imprecision

**Figure 3:**
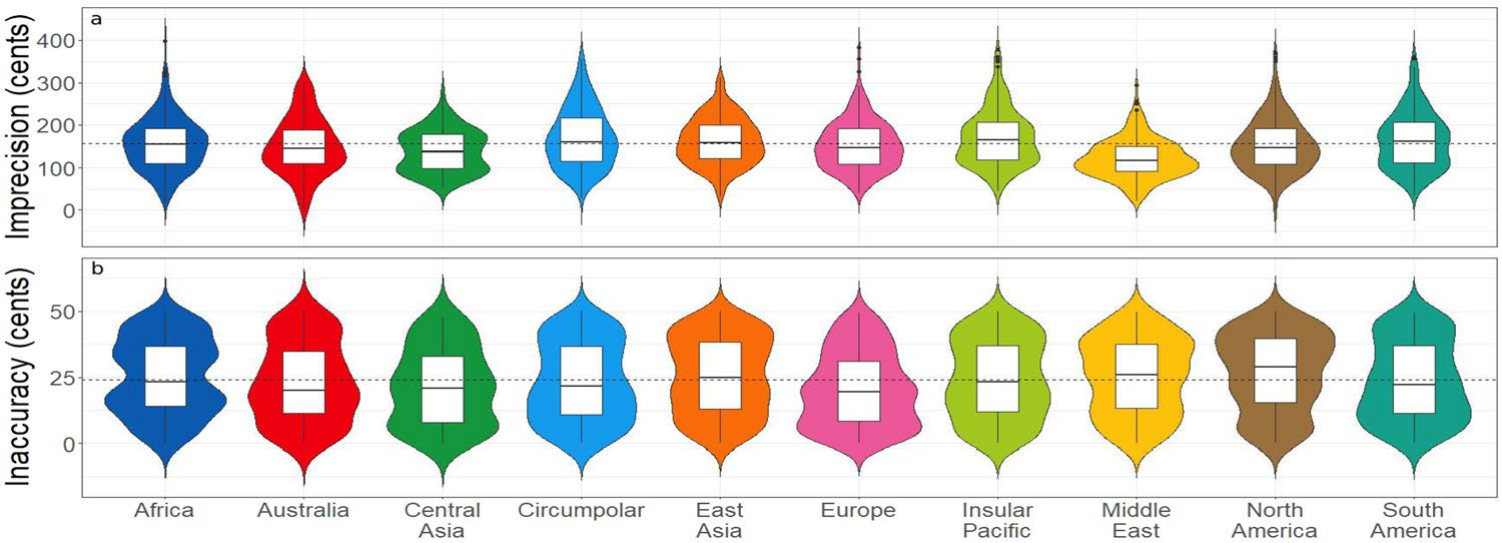
Global vocal pitch-class imprecision and inaccuracy. (**a**) The distributions of pitch-class range across regions as a measure of pitch-class imprecision, shown as violin plots. (**b**) The distributions of pitch-class accuracy across regions, shown as violin plots. The violin plots are color-coded for musical style-region as per Fig. 1. The outer colored plot presents a mirrored kernel density estimation, meaning that the shape on either side of the box plot is a smoothed histogram of the data. The inner box plot presents the median as a horizontal line, the interquartile range (IQR; 25th and 75th percentiles) as a box, and whiskers that extend to the maximum and minimum values (excluding outlying points, which are plotted individually. Outlying points are those that are further than 1.5 times the IQR from either the 25^th^ or 75^th^ percentile.) The global mean for each variable is represented by a dashed line.

The mean global pitch-class imprecision, defined as the mean pitch-class range across the 418 songs, was 155 cents (SD = 62.2, slight right skew = 0.59). The mean values regionally spanned from 121 cents in the Middle East-North Africa to 170 cents in the Insular Pacific and Circumpolar regions. Hence, no world region showed an imprecision in vocal production less than 1 semitone, with the global mean being just beyond 1.5 semitones. The distributions of the pitch-class ranges, as shown in Fig. 3a, differed across regions. Central Asia, the Middle East-North Africa, and the Insular Pacific had seemingly bimodal distributions, whereas the Circumpolar region had an especially wide and flat distribution.

Due to the non-normality (Shapiro–Wilk’s test, W = 0.98, p < 0.001) and non-homogeneity of variance (Levene’s test, F = 6.78, df = 9, p < 0.01) in the data, we tested for differences in imprecision between world regions using a multiple-comparisons method that is robust to non-heteroscedasticity and that controls the family-wise error rate^33^. The results revealed several significant regional differences. Pitch-class imprecision was (a) lower in the Middle East-North Africa region than all other regions (p < 0.001), except Central Asia; (b) lower in Central Asia than the Circumpolar and Insular Pacific regions (p < 0.001), as well as East Asia and South America (p < 0.01); (c) higher in the Insular Pacific region than Europe (p < 0.01) and North America (p < 0.05); and (d) higher in the Circumpolar region than Europe (p < 0.05). No other comparisons were significant. To summarize, songs from the Middle East-North Africa region, and to a lesser extent Central Asia, demonstrated lower-than-average pitch-class imprecision, whereas songs from the Insular Pacific, and to a lesser extent Circumpolar region, demonstrated higher-than-average pitch-class imprecision. Similar results for pitch-class imprecision were found using the inter-quartile range: songs from the Middle East-North Africa region were the most precise, whereas songs from the Circumpolar region, Insular Pacific and South America were the least precise.

#### Inaccuracy

Figure 3b demonstrates the global pattern of pitch-class inaccuracy. The average global pitch-class inaccuracy was 24 cents (SD = 14.5), which is indicative of a generally random pattern of tuning compared to the mathematical template (i.e., 12-ET). This pattern was consistent across regions. An analysis using the same multiple-comparisons method as above revealed that pitch-class inaccuracy was significantly lower in Europe than in North America (p < 0.001), Africa, and East Asia (p < 0.01), and the Middle East-North Africa (p < 0.05). However, no other comparisons were significant. Another way to evaluate inaccuracy is to compare the *variance* of the inaccuracy values, not just the values themselves. Songs that are well-tuned to 12-ET, such as European art songs, should include pitch-classes with *consistently* low inaccuracy values, demonstrating low inaccuracy *and* low inaccuracy variance. Songs that are not tuned to 12-ET should produce pitch-classes with a random distribution (from 0 to 50 cents) of inaccuracy values, leading to a high variance for inaccuracy, though not necessarily a high average inaccuracy. The variance of inaccuracy values across regions was compared using multiple pairwise Levene tests with Bonferroni correction. The results showed that the variance of pitch-class inaccuracy was not significantly different among regions. A general Levene test also showed no heterogeneity (F = 0.47, df = 9, p = 0.89). The distributions of pitch-class inaccuracy, as seen in Fig. 3b, are also of note. The bimodal distribution of Africa is particularly interesting, as is the nearly reciprocal relationship between the distributions of North and South America. To summarize, these results suggest that *a cappella* traditional songs are generally not sung in 12-ET, although European folk songs are more accurate to that tuning system than are songs from other global regions.

### Study 2: instrumental pitch production

#### Imprecision

**Figure 4:**
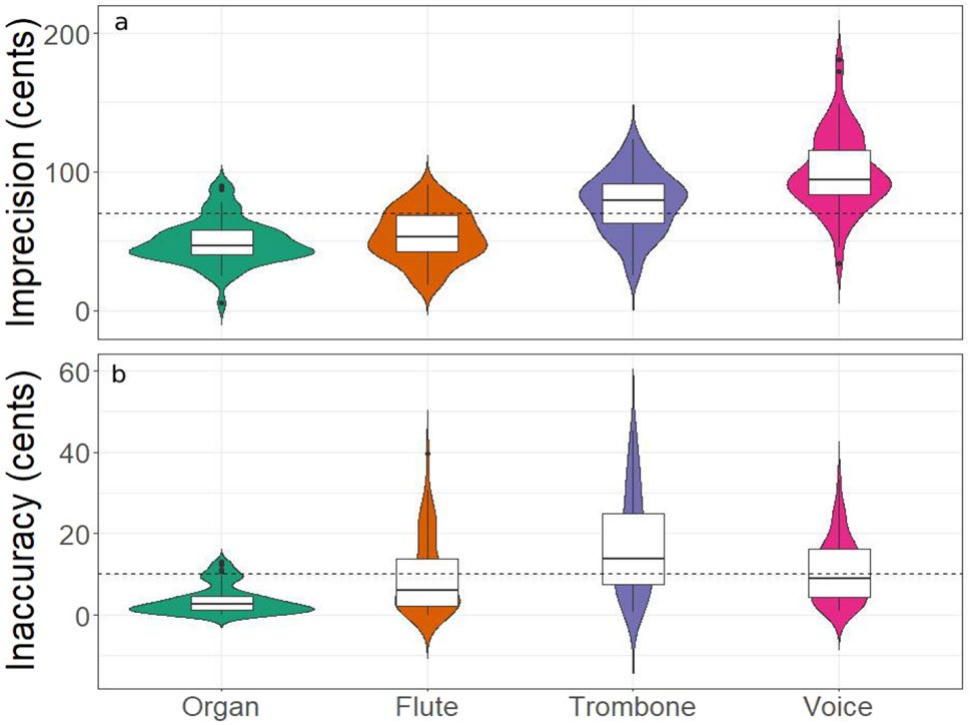
Instrumental pitch-class imprecision and inaccuracy. (**a**) The distributions of pitch-class range across instruments as a measure of pitch-class imprecision, shown as violin plots. (**b**) The distributions of pitch-class inaccuracy across instruments, shown as violin plots. The violin plots have the same properties as described in the legend of Fig. 3. The mean of each variable is represented by a dashed line.

Figure 4a presents the distributions of pitch-class imprecision across the instruments studied. The expected trend of organ < flute < trombone < voice was generally observed. Due to the non-homogeneity of variance in the data (Levene’s test, p < 0.01), we tested for differences between instruments using the same multiple-comparisons method as above. The pitch-class imprecision for each instrument differed significantly from every other instrument (p < 0.001), except for the organ and flute, which did not differ (p = 0.5).

#### Inaccuracy

Figure 4b shows the distributions of pitch-class inaccuracy across the instruments studied. The expected trend for pitch-class inaccuracy (organ < flute < trombone < voice) was not observed, mainly because the voice was more accurate than predicted. Due to the non-normality (Shapiro–Wilk’s test, p < 0.01) and non-homogeneity of variance (Levene’s test, p < 0.01) in the data, we tested for differences between instruments using the same multiple-comparisons method as above. The pitch-class inaccuracy of the organ was significantly lower than every other instrument (p < 0.001). The pitch-class inaccuracy of the trombone was significantly higher than that of the flute (p < 0.005) and voice (p < 0.05), but the inaccuracy of the flute and voice did not differ significantly (p > 0.5). As above, we compared the variance of the inaccuracy values across instruments using multiple pairwise Levene tests with Bonferroni correction. The organ had significantly lower variance in pitch-class inaccuracy than all of the other instruments. The flute did not differ significantly from either the voice or the trombone. However, the voice showed a significantly lower inaccuracy variance than the trombone. These results generally support the previous conclusion: the organ was the most accurately tuned, whereas the trombone was the least accurately tuned. In summary, the results for both pitch-class imprecision and inaccuracy mostly followed the expected trends, except that the singer was more accurate than expected compared to the trombone.

### Imprecision/inaccuracy correlations

**Figure 5:**
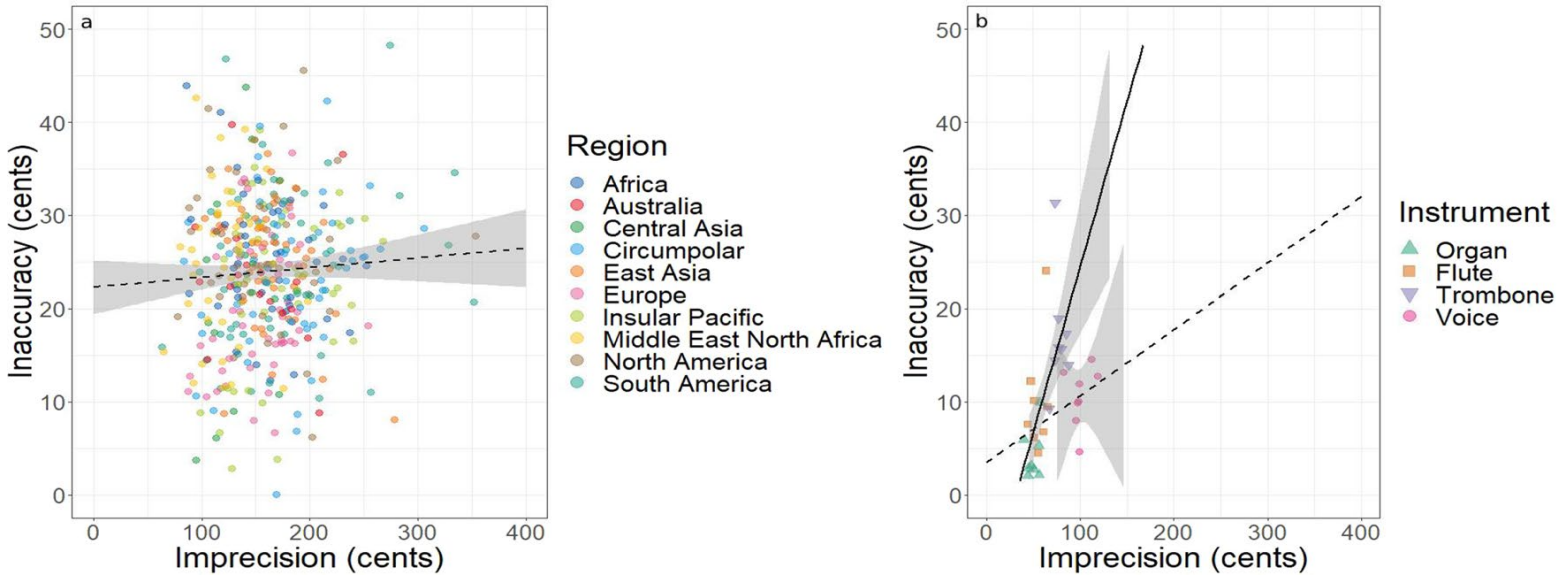
The relationship between imprecision and inaccuracy in each of the two studies. (**a**) Global vocal pitch production across regions in Study 1, taking the mean pitch-class inaccuracy for each song relative to the same song’s mean pitch-class imprecision. The dashed line indicates the linear regression (F = 1.435, df = 415, p > 0.2; adjusted R^2^ = 0.001) of vocal imprecision and inaccuracy across all regions, with the shaded grey region indicating the standard error. (**b**) Pitch production profiles across instruments in Study 2, taking the average pitch-class inaccuracy for each song relative to the same song’s average pitch-class imprecision. The dashed line indicates the linear regression for the voice alone (F = 0.392, df = 6, p > 0.5, adjusted R^2^ = 0.095), while the solid line indicates the linear regression for the three non-vocal instruments combined, with the shaded grey region indicating the standard error (F = 18.41, df = 22, p < 0.001, adjusted R^2^ = 0.431).

Figure 5 examines correlations between pitch-class imprecision (on the x axis) and pitch-class inaccuracy (on the y axis) for both studies. Figure 5a demonstrates that vocal pitch-class imprecision and inaccuracy were relatively uncorrelated in the global ethnographic sample from Study 1, and that there were no clear regional clusters. The global Pearson’s correlation between these two variables was 0.06 (t = 1.20, df = 415, p = 0.23, dashed line in Fig. 5a). The correlation was strongest in East Asia (r = − 0.31), Africa (r = − 0.30), and Central Asia (r = 0.30), and weakest in the Middle East-North Africa (r = 0.08).

Figure 5b shows the correlations for Study 2, and provides further support for the observation that vocal pitch-class imprecision and inaccuracy are only weakly correlated, even for the trained Western singer (Pearson’s correlation = 0.25, t = 2.29, df = 6, p = 0.55), and especially compared to the fixed-pitch instruments. The overall correlation between pitch-class imprecision and inaccuracy in Study 2 was 0.39 (t = 2.29, df = 30, p = 0.03). But when the voice data was removed, the correlation between instrumental pitch-class imprecision and inaccuracy was 0.67 (t = 4.29, df = 22, p < 0.001). Unlike in Study 1, relatively clear clusters are present in the data from Study 2, suggesting that the relationship between pitch-class imprecision and inaccuracy can be used as an instrumental profile. Fixed-pitch instruments (including the organ and flute) are both accurate and precise, whereas variable-pitch instruments (including the trombone) are both less precise and less accurate. In contrast, the voice is relatively imprecise, regardless of the singer’s accuracy.

## Discussion

In the present study, we carried out the first analysis of vocal imprecision and inaccuracy in a global corpus of indigenous vocal songs, covering the principal musical-style regions of the world, as based on Lomax’s seminal work^28^. The study moves beyond the circularity of tunable instruments being engineered to produce music according to mathematically-specified templates, and instead investigates the actual pitch properties of indigenous vocal songs. The main take-aways of our analyses are clear. Study 1 indicated that vocal imprecision in sung pitch production is universal, with the range of vocal pitch-classes averaging just beyond 1.5 semitones worldwide. In addition, it demonstrated that our sample of indigenous and traditional music did not conform to the mathematical predictions of 12-ET. Study 2 supported the observation that vocal imprecision is universal, although it also showed that a highly trained singer can accurately conform to a given tuning system. Study 2 also showed that the pitch-class imprecision and inaccuracy of instruments seem to vary systematically according to their degree of pre-tuning, where the voice is both the least tunable and the least precise instrument studied here. As such, these results provide strong evidence for universal vocal imprecision and a dearth of evidence for mathematically-based vocal tuning worldwide, which collectively support physiology-based models of music evolution like the Interval Spacing theory^5,6^. The results also indicate that future models of scale structure, beyond proposing universal physiological mechanisms, must also account for the enculturating effects that long-term musical training may exert on vocal physiology, especially with regard to tuning accuracy.

The results of the present study point to a fundamental and universal constraint that has shaped the nature of musical scales from their probable origin in vocal music. Harmonicity theory cannot account for either the number of tones in a scale or the spacing between successive scale tones. Previous cross-cultural analyses have demonstrated a preference for scales having 5–7 tones per octave and a predominant spacing of a whole tone between adjacent scale tones^21,30^. In Study 1, we found a global pitch-class imprecision of 155 cents (just greater than 1.5 semitones). Scale-steps smaller than this would cause adjacent pitch-class distributions to overlap, leading to a reduction in pitch distinguishability and musical communication. Therefore, if scale tones have a tendency to be at least 1.5 semitones apart on average, then this constrains scales to have fewer than 8 pitch-classes per octave (1200 cents in an octave / 155 cents of vocal imprecision = 7.8 pitch-classes). Likewise, a pitch-class imprecision of 155 cents creates a driving force to distance successive scale pitches by steps closer to 200 cents (a whole tone) than 100 cents (a semitone) or less, which jibes with the observed cross-cultural trends. In other words, our results provide a physiological rationale for why *semitones* are considered to be the smallest scale interval in world musics, a phenomenon that is in no way explainable by Harmonicity theory.

While our analysis of the typological properties of the scales in this corpus will be presented elsewhere, we report here that the mean intervallic spacing between successive pitches in the 418 scales was 2.2 semitones, even larger than the 1.5-semitone imprecision in pitch-classes. This raises the important point that the true constraint on scale structure might not stem from the imprecision of pitch-classes per se, but instead the imprecision of *interval*-classes, since intervals define the actual pitch movement of the voice during melody production. As mentioned in the Introduction, scales are an abstraction of melodies, not the other way around^17^. We are currently in the process of developing a computational method for analyzing interval-class imprecision. It is quite likely, as per the results of Pfordresher and Brown^5^, that interval-class imprecision for this sample would exceed 1.5 semitones and be more in line with cross-cultural trends for the spacing between successive scale tones.

We observed some cross-cultural variation in the pitch-class imprecision data (see Fig. 3a), spanning from a lower mean value of 121 cents in cultures that possess a priori scale theories (the Middle East-North Africa and Central Asia) to a higher value of 170 cents in more-indigenous cultures from the Circumpolar region and the Insular Pacific (e.g., the indigenous Taiwanese populations). This suggests that a certain degree of cultural mediation is superimposed upon the presumed physiological constraint on vocal precision, a supposition that is further supported by the results with the trained Western singer in Study 2 (Fig. 4a). This apparent enculturation effect bears further study.

The claim that scales should have 7 or fewer pitches per octave and favor successive intervals of a whole tone or larger does not, by itself, specify the actual sequences of steps that comprise scales worldwide. In fact, we have observed immense scale diversity both within and between cultures, which suggests that humans can combinatorially create a near-infinite variety of scale sequences, even when constrained by vocal interval-spacing considerations. From the standpoint of the current discussion, it will be important to examine if differences in pitch-class imprecision between the cultures mentioned above are correlated with differences in scale properties. We address this issue in our forthcoming typological analysis of the scales in the corpus.

Pitch-class inaccuracy demonstrated a much more uniform trend across cultures than imprecision, with a mean value of 24 cents. The plots in Fig. 3b revealed that inaccuracy values spanned the full extent of the 50-cent bin, suggesting a random pattern with regard to the mathematical template. In addition, we found little evidence of cross-cultural variation in inaccuracy, although Europe—the source of 12-ET—showed slightly lower inaccuracy than the rest of the world. This brings into question a key assumption of tuning theories based on a priori mathematical principles. Vocal music does not seem to show a concern for precise mathematical ratios, which is perhaps not surprising given the “island” nature of pitch-classes (the current study) and interval-classes^5^ in vocal music. Overall, imprecision seems to be a stronger constraint in the formation of scale structure than inaccuracy. Despite all of the attention given to perfect ratios in academic accounts of scales, mathematical tuning does not seem to be a critical factor in vocal scales cross-culturally. Scales seem to show flexibility with regard to perfect ratios, especially in indigenous cultures and especially in vocal music. It is important to state that, for cultures that lack literary texts about a priori tuning theories, we have no idea what the actual pitches *should* be in their music. The best we can do is examine the sung pitches against a mathematical template and determine the extent to which the pitches conform with it. The current study, using the tuning protocol of 12-ET as its template, showed a lack of regard for this tuning system in all world regions.

Although this is, to our knowledge, the largest and most diverse analysis of global song thus far researched, it has a number of key limitations. First, very few recordings of solo traditional songs were obtainable for several world regions. For example, it was difficult to find samples from Central Asia, and especially India, since most vocal recordings from this latter region are of the classical tradition. Defining indigenous and folk musical traditions, in contrast to art and classical traditions, as well as delimiting the geographical regions of various cultures, was generally a challenge for this study. This approach cannot account for within-culture diversity, cultural blending due to migration, and other nuances of cultural identity. Other significant stylistic regions that were not sampled in this study included Melanesia and Polynesia, which we aim to sample in future work.

One of the main advantages of the current project was not only the global sampling of the vocal songs, but the use of Lomax’s 1968 Cantometric map to define the regions of song style with musicological rigor. However, there were limitations in this approach. For example, Lomax’s “Old High Culture” designation was too broad for our purposes, forcing us to segment it into three sub-regions (the Middle East-North Africa, Central Asia, and East Asia). Another limitation is that Lomax revised his own classification scheme in a 1980 book chapter^34^, creating points of difference from the map that we used. For example, Europe became subdivided into Western Europe and Central Europe. In addition, new designations, such as Proto-Melanesia and Nuclear America, were added to the scheme. But perhaps the most fundamental limitation of our current approach to the musical map is that a 10-region division of world musical styles is very coarse-grained in light of the hundreds if not thousands of musical styles that no doubt exist. Therefore, future research in this area, even that restricted to indigenous samples, will need to work towards finer-grained classification of musical styles than was attempted here. However, such classification must be based on well-researched musicological analyses, such as Lomax’s.

Next, there were limitations inherent to our analytical methods. Any qualitative study is prone to subjectivity, and this is especially true for by-ear analyses of non-Western music by Western musicians. Although we consider our main quantitative results to be objective, we ultimately defined the pitch-class boundaries by hand. Three analysts worked in parallel on a subset of the songs, reviewed them together, and then reanalyzed them before moving on to the next subset, and the results of our analyses were indeed consistent across multiple interim checks over roughly a year’s time-span. Still, our particular analysis of each piece is likely irreplicable, as the precise location of pitch-class boundaries might change depending on the analyst—or even within a single analyst—based on their attention, the number of times they have listened to the piece, and what they listened to (and looked at) directly prior. Our pitch-class segmentation method relied on visualization of the melographs, which we found to be the most reliable approach. However, visualization introduces its own biases by forcing the analyst to interpret a song in a way that they might not have done so through listening alone. Such analytical issues pose significant challenges to any work on vocal music, and are the subject of vigorous discussion in computational musicology and an ongoing international collaboration investigating expert disagreement in song transcription^35^.

Ongoing work from our lab is also continuing to refine the computational methods presented here. For example, although we used Tarsos for pitch analysis in this study, the use of Tony and its pYIN pitch-tracker^36,37^ may provide better fundamental frequency annotations. In addition, if both lower and upper boundaries were defined for each pitch-class, rather than a single intervening boundary, then each pitch-class range could be calculated with greater accuracy. This is especially true when the ranges of adjacent pitch-classes overlap. This updated approach would allow for the degree of that overlap to be explicitly measured. In addition, we plan to test the reliability of our precision data against other measures of variability that are less sensitive than range to extreme values, such as kurtosis and the standard deviation. Lastly, while we used 12-ET as our mathematical template for calculating pitch-class inaccuracy, we are currently examining other mathematical tuning systems^39^, including just intonation and equidistant divisions of the octave beyond 12-ET. Although 12-ET has generally been treated as interchangeable with just intonation for measuring pitch accuracy, largely due 12-ET’s relative simplicity as a cent-based template, evaluating inaccuracy relative to just intonation would allow us to better evaluate the claims that ratio-based or other harmonic tuning principles are cross-culturally universal.

In addition, although Study 2 confirmed that tuning fixedness influences pitch-class properties that are relevant to scale construction, its scope was purely exploratory. Future studies will aim to recruit multiple musicians per instrument, instruct the musicians to play every piece multiple times, examine performance reliability across more than one session, and cover a wider range of instruments, especially cross-culturally. This will allow for the examination of within-musician and within-instrument variability, both short- and long-term. We would expect that these variables would be associated with the instrument’s tuning fixedness and that they would follow the same trends of pitch-class imprecision and inaccuracy that were observed in the present study. That is, the organ would be the least variable while the voice would be the most variable.

In tandem, the results of these studies suggest that Interval Spacing theory might serve as a general bottom-up theory of how pitch-production constraints shape scale structure *across instruments* and therefore across the evolution of musical scales. We suggest that the structure of the earliest scales was driven by the constraints of the most universal pitch-production mechanism, namely the solo voice. The later evolution of melodic musical instruments and the even later formulation of mathematical tuning theories for the engineering of instruments with specific tuning properties allowed instruments to be pre-tuned in a way that the voice cannot be. This shifted the focus for musical scales from the bottom-up physiological constraints intrinsic to the voice to the top-down theoretical considerations underlying the practice of instrument design in musically literate cultures. We argue that the former is a far better model of the origin of musical scales than the latter.

Lastly, while our focus has been on constraints at the level of production, it is worth noting that perceptual constraints may have offered various affordances for scale structure over the course of scale evolution. For example, the need to entrain with others, and/or to harmonize with them, may have encouraged the use of different musical textures. The harmonies emerging from these textures may have shaped musical scales beyond the simple melody-based forms that we studied here. Hence, we view Interval Spacing theory as a fundamental theory of the origin of musical scales, one that can be used to build models of musical tonality^40^ and the cultural evolution of music^41,42^.

Overall, the study has provided a novel approach to investigating the evolution of musical scales. Instead of starting with the top-down mathematical principle that scales optimize their structure according to harmonic ratios, it has looked to bottom-up physiological mechanisms of vocal production. Likewise, instead of looking at melodies produced by tunable instruments such as chordophones, the study has examined ethnographic field recordings of vocal music from indigenous cultures, doing so from all of the major musical-style regions of the world. The results revealed a universal propensity for singers to produce pitches in an imprecise manner, with a pitch range of around 1.5 semitones, regardless of culture. Such imprecision provides a significant constraint on the formation of musical scales by simultaneously providing a lower limit on the spacing between adjacent scale tones and an upper limit on the number of scale tones within an octave. They also provide a physiological rationale for why semitones are considered to be the smallest scale interval in world musics. Observations such as these underlie the Interval Spacing model of the origin of musical scales, which posits that scales evolved to optimize their structure according to the physiological limitations of the earliest human music, namely song^5,6^. Our companion analysis will look at the intervallic and structural properties of the 418 scales. Future work should aim to identify the physiological basis of pitch-class imprecision and inaccuracy, and posit mechanisms underlying the refinement of vocal control that can occur in various forms of musical training, expertise, and enculturation.

## Data Availability

All materials for this study are available at the following public Open Science Framework repository: https://osf.io/jn78c/?view_only=89846926c138478dad2990e833fb5e38.
